# Whole-Transcriptome Analysis of Verocytotoxigenic *Escherichia coli* O157:H7 (Sakai) Suggests Plant-Species-Specific Metabolic Responses on Exposure to Spinach and Lettuce Extracts

**DOI:** 10.3389/fmicb.2016.01088

**Published:** 2016-07-12

**Authors:** Louise Crozier, Pete E. Hedley, Jenny Morris, Carol Wagstaff, Simon C. Andrews, Ian Toth, Robert W. Jackson, Nicola J. Holden

**Affiliations:** ^1^Cell and Molecular Sciences, The James Hutton InstituteDundee, UK; ^2^School of Chemistry, Food and Pharmacy, The University of ReadingReading, UK; ^3^School of Biological Sciences, The University of ReadingReading, UK

**Keywords:** DNA microarray, stress response, *E. coli* O157:H7, vegetables, leaves, roots, biological, adaptation

## Abstract

Verocytotoxigenic *Escherichia coli* (VTEC) can contaminate crop plants, potentially using them as secondary hosts, which can lead to food-borne infection. Currently, little is known about the influence of the specific plant species on the success of bacterial colonization. As such, we compared the ability of the VTEC strain, *E. coli* O157:H7 ‘Sakai,’ to colonize the roots and leaves of four leafy vegetables: spinach (*Spinacia oleracea*), lettuce (*Lactuca sativa*), vining green pea (*Pisum sativum*), and prickly lettuce (*Lactuca serriola*), a wild relative of domesticated lettuce. Also, to determine the drivers of the initial response on interaction with plant tissue, the whole transcriptome of *E. coli* O157:H7 Sakai was analyzed following exposure to plant extracts of varying complexity (spinach leaf lysates or root exudates, and leaf cell wall polysaccharides from spinach or lettuce). Plant extracts were used to reduce heterogeneity inherent in plant–microbe interactions and remove the effect of plant immunity. This dual approach provided information on the initial adaptive response of *E. coli* O157:H7 Sakai to the plant environment together with the influence of the living plant during bacterial establishment and colonization. Results showed that both the plant tissue type and the plant species strongly influence the short-term (1 h) transcriptional response to extracts as well as longer-term (10 days) plant colonization or persistence. We show that propagation temperature (37 vs. 18°C) has a major impact on the expression profile and therefore pre-adaptation of bacteria to a plant-relevant temperature is necessary to avoid misleading temperature-dependent wholescale gene-expression changes in response to plant material. For each of the plant extracts tested, the largest group of (annotated) differentially regulated genes were associated with metabolism. However, large-scale differences in the metabolic and biosynthetic pathways between treatment types indicate specificity in substrate utilization. Induction of stress-response genes reflected the apparent physiological status of the bacterial genes in each extract, as a result of glutamate-dependent acid resistance, nutrient stress, or translational stalling. A large proportion of differentially regulated genes are uncharacterized (annotated as hypothetical), which could indicate yet to be described functional roles associated with plant interaction for *E. coli* O157:H7 Sakai.

## Introduction

Verocytotoxigenic *Escherichia coli* (VTEC) comprise an important group of food-borne pathogens that can enter the human food chain from contaminated plant as well as meat products. It is estimated that ∼20–25% of food-borne VTEC outbreaks worldwide arise from contaminated crop plants, based on publicly available reports (Greig and Ravel, 2009). Plant-based foods that carry the highest risk are leafy greens eaten raw as salads, and include foodstuff consumed raw or lightly cooked, i.e., fruits, vegetables, and sprouted seeds (EFSA Panel on Biological Hazards (BIOHAZ), 2013). It is now established that pathogenic *E. coli* can interact with plants and use them as secondary hosts (Holden et al., 2015). However, there are still many questions over the mechanism of plant adaptation and, in particular, the role of bacterial-stress responses in plant colonization. The main reservoir for VTEC is ruminants where regular fecal-shedding leads to bacterial dispersal into the environment, necessitating adaptation for survival and persistence and the prevailing view is that exposure to environments outwith the primary reservoir induces metabolic and physio-chemical stresses. However, the prevalence of certain *E. coli* isolates in the wider environment (Ishii et al., 2009; Brennan et al., 2010), including on plants, suggests that these bacteria do not simply survive and persist on plants, but instead have evolved into semi-specialized plant colonizers to facilitate persistence in the environment. Mesophilic species such as *E. coli* are adapted to proliferate over the range of temperatures encountered in the wider environment (Ratkowsky et al., 1982) given sufficient nutrients. It appears that VTEC belongs to a group of *E. coli* isolates that have evolved to adapt to a lifestyle that at least partly involves association with plants, and so can use them as secondary hosts (Holden et al., 2009). Therefore, a better understanding of the bacterial response to plants as hosts will help to improve our perspective of VTEC as a plant-borne human pathogen and thus inform on risk analysis and mitigation strategies.

Global-transcriptomic analysis has identified a range of responses (e.g., induction of stress-resistance) of pathogenic and non-pathogenic *E. coli* to various plant-associated environments (Kyle et al., 2010; Fink et al., 2012; Hou et al., 2012, 2013; Landstorfer et al., 2014; Linden et al., 2016). However, in many reports on plant-colonization transcriptomics the bacteria were initially cultured at body temperature (37°C) and were subsequently exposed to plant (or plant extracts) at environmental temperature (∼18°C); such experimental regimes result in a considerable temperature shift, in addition to the exposure to plant or plant extracts (Thilmony et al., 2006; Kyle et al., 2010; Hou et al., 2012, 2013; Jayaraman et al., 2014). In other reports, the entire experiment was performed at 37°C (Bergholz et al., 2009; Fink et al., 2012; Visvalingam et al., 2013; Landstorfer et al., 2014) rather than at a temperature (i.e., ∼18°C) relevant to plants growing in temperate zones. Temperature-dependent control of gene expression in *E. coli* and other bacteria is well-characterized (Phadtare and Inouye, 2008) and it is clear that temperature-induced global expression changes can obscure or complicate responses to other stimuli (Polissi et al., 2003; King et al., 2014). Thus, the specific reaction to the plant might not be accurately distinguished in previous reports where inappropriate temperature regimes were imposed.

Here, we investigate adaptation to and colonization of leafy salad plants by the predominant VTEC serotype O157:H7, using techniques for cultivable bacteria. We assess changes in gene expression profile of *E. coli* O157:H7 (isolate Sakai) at an environmentally relevant temperature to negate any temperature-dependent responses. Expression responses to a range of plant extracts of varying complexity were tested to avoid any host-defense influences, allowing a clearer identification of the other drivers of the bacterial response. In addition, use of extracts is expected to reduce the heterogeneity imposed on bacterial population by propagation on living plants (as observed for individual gene expression *in planta* (Rossez et al., 2014a). Spinach was selected as the focus for the response analysis because there have been a number of reported VTEC outbreaks from spinach. Lettuce was included as a comparison for the response to cell wall polysaccharides (CWPSs) as there have also been lettuce-associated VTEC outbreaks and our previous data showed differences in the adherence interactions (Rossez et al., 2014a). We focus on early expression responses (prior to proliferation), to minimize cell-division-dependent gene expression changes. This approach thus considers expression change during the initial, adaptive interactions that occur before establishment. The hypothesis tested is that *E. coli* O157:H7 undergoes adaptive changes in gene expression upon exposure to the plant that affects the outcome of colonization and persistence. We expect gene expression changes to be quite distinct from those reported during ruminant colonization (Dahan et al., 2004). Whole transcriptome analysis was coupled with investigation of *E. coli* O157:H7 growth potential over short-time scales in plant extracts and longer-term on plant hosts. The findings relayed here support the notion that plants are genuine secondary hosts for VTEC, rather than incidental habitats.

## Results

### *E. coli* O157:H7 Exhibits Major Differences in Global Expression in Response to Growth at 37 or 18°C

We hypothesized that some VTEC isolates undergo adaptive gene expression changes that enable them to colonize plants. In order to gain insight into the mechanisms of adaptation to the plant and to discern any tissue or plant species-associated differences that may occur, transcriptional changes exhibited by *E. coli* O157:H7 (Sakai) were examined following exposure to plant extracts. The initial stages of the plant–bacterium interaction were examined by whole-transcriptome analysis, using established *E. coli* DNA microarray technology. For this purpose, *E. coli* O157:H7 (Sakai) was cultured at a plant-relevant temperature (18°C) prior to, and during, exposure to plant extract. However, in order to determine the impact of incubation temperature on the transcriptome, it was necessary to firstly compare global gene expression for cultures maintained in minimal M9 glycerol medium at 18°C (both pre- and post-culture) and 37°C. Both cultures were transferred to fresh medium at their respective temperatures for 1 h prior to sampling, representative of late lag to early exponential phase. The regime employed ensured assessment of temperature-dependent growth, avoiding any temperature shift or shock effects.

**FIGURE 1:**
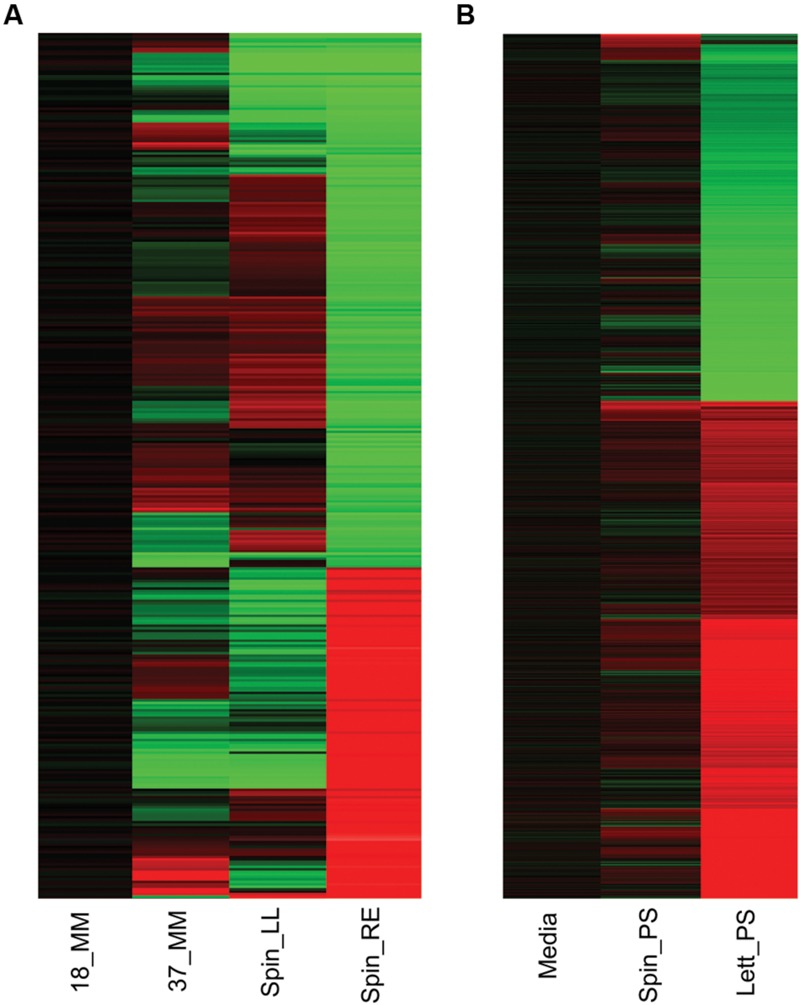
**Gene expression overview.** Heatmap of *Escherichia coli* O157:H7 (Sakai) total gene expression changes in response to different temperature and plant extract treatments. Changes in gene expression for *E. coli* O157:H7 (Sakai) grown for 1 h at 18°C are compared to cultures grown similarly at 37°C (37_MM), or at 18°C containing spinach (*S. olercera*) extracts of leaf lysates (Spin_LL) or root exudates (Spin_RE; **A**). Changes in gene expression for exposure to 1 h exposure to medium at 18°C containing polysaccharide extracts from spinach (*S. olercera*; Spin_PS) or lettuce (*Lactuca sativa*; Lett_PS) are compared to a baseline for *E. coli* O157:H7 (Sakai) in medium containing a no-plant control extract (‘Media’; **B**). Significant changes in expression of at least twofold are shown for induced (red) or repressed (green) genes.

As expected, gene expression of *E. coli* O157:H7 (Sakai) grown for 1 h at 18°C was markedly different from that of the culture grown at 37°C (**Figure 1**). A total of 1,127 genes were differentially expressed in response to incubation temperature, representing 20.6% of *E. coli* O157:H7 Sakai ORFs. Of these, 500 genes were induced and 627 genes (9.16 and 11.48% of Sakai ORFs) were downregulated (Supplementary Table S1). Notable changes in expression of specific genes at 18°C (cf. 37°C) included repression of a subset of genes in the locus of enterocycte effacement (LEE). These included *ler* (130-fold repression; which encodes the master regulator of the *lee* genes), several type III secretion (T3SS) genes (ECs4583, *escC, escJ, escS*, and *espF*: repressed by 15-, 10-, 12-, 20-, and 10-fold respectively; Supplementary Table S1). The control of *ler* expression by low temperature is likely caused by H-NS silencing, which is known to suppress A/E lesion formation below 37°C (Umanski et al., 2002). Motility genes were also repressed, particularly in the *flg* and *fli* loci (e.g., *flgBCDE*, 26–59-fold repressed; *fliE*, 26-fold repressed. Three hypothetical genes in an apparent operon of unknown function (ECs2623-2625) were amongst those most strongly repressed (∼200-fold), as were a series of prophage CP-933T genes (*coxT*, Z2971-4; 46–276-fold) possibly in response to QseA control (Kendall et al., 2010). The major class of genes subject to induction at 18°C were those involved in various aspects of stress resistance: acid resistance (e.g., ECs2098, *gadABCE*; 38–121-fold induced), heavy-metal resistance (e.g., *cusBX*; 56–81-fold induced), putrescine metabolism (e.g., *ygjG*, ECs3955; 52–65-fold induced), multidrug eﬄux (e.g., *sugE*; 33-fold induced) and osmotic stress (*proVW*; ∼25-fold). In addition, a cluster of genes (ECs1653-1655; 14–28-fold) of unknown function was strongly induced as were several genes involved in biofilm formation (Z2229, ECs2085, *bdm*, c_1914; 51–59-fold; Supplementary Table S1). In summary, the expression data suggest that growth at ambient rather than body temperature causes reduces motility and increases sessile behavior, reduced ability to colonize the mammalian gut and suppresses some prophage, but raises ability to resist a range of environmental stresses (a possible adaptation to slower growth at lower temperature). Such temperature-dependent changes would be expected to confound interpretation of expression data obtained in previous studies on bacterial plant colonization where a temperature change was included along with plant exposure – a complication that was avoided within the research reported below.

### Exposure of *E. coli* O157:H7 to Different Plant Extracts Elicits Distinct, Major Alterations in Global-Gene Expression

The whole transcriptome of *E. coli* O157:H7 (Sakai) was subsequently examined during the early stages of the plant interaction, under the conditions (18°C, 1 h) employed above. Extracts of spinach (*Spinacia oleracea*) and roundhead lettuce (*Lactuca sativa*) were used as these have been associated with large-scale food borne outbreaks of VTEC previously (Cooley et al., 2007; Friesema et al., 2008). Leaf lysates (spinach) represent the combined cellular material and apoplast; root exudates (spinach) represent plant root-derived substrates; and leaf CWPSs (derived from spinach or lettuce) represent the cell wall components that include molecules involved in plant–microbe interactions. To provide an indication of any species-specific expression differences, a leaf CWPS extract from lettuce (*L. sativa*) was used to compare with that from spinach. CWPSs are the least complex of the plant extracts employed here and so are expected to induce more modest expression changes than the other plant samples, which should facilitate identification of any species differences that might occur.

**FIGURE 2:**
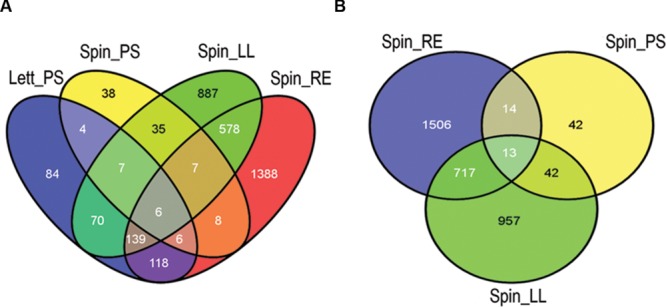
**Differentially expressed gene comparison by plant species and tissue.** Overview of common and specific *E. coli* O157:H7 (Sakai) genes differentially expressed in plant extracts. The number of genes was compared in a Venn diagram of all four treatments **(A)** and for the spinach extracts only **(B)**. Key: ‘Spin,’ spinach (*S. olercera*); ‘Lett,’ lettuce (*L. sativa*); ‘LL,’ leaf lysates; ‘RE,’ root exudates; ‘PS,’ cell wall polysaccharides (CWPSs). Images were generated using the Venny program (Oliveros, 2007).

Wholescale changes in *E. coli* O157:H7 (Sakai) gene expression occurred following 1 h of exposure to the different extracts at 18°C (**Figure 1**). Exposure to spinach leaf lysate resulted in differential expression of 27% of the Sakai genome, 745 genes were induced and 738 were repressed, while 35% of the Sakai genome was differentially expressed on exposure to spinach root exudates: 981 induced and 972 repressed. In general, there appeared to be an inverse correlation in differential gene expression between exposure to spinach root exudates and spinach leaf lysates (**Figure 1**). The response to leaf CWPSs was examined to exclude the effects of other leaf components (e.g., apoplastic fluid and intracellular contents). Gene expression for *E. coli* O157:H7 (Sakai) exposed to spinach and lettuce CWPS for 1 h showed marked differences between species, with 460 and 97 genes displaying differential expression in response to lettuce and spinach CWPS, respectively, when compared to the response to the negative, no-plant control (an extract prepared from vermiculite, the inert plant growth substrate; **Figure 1**). Thus, the extent of expression change was far less with the CWPS (average of 3.2%) than with the leaf and root samples (average of 13%), as anticipated. Comparison between the species or tissue types showed little commonality in differentially expressed genes (**Figure 2**). This is well-illustrated by the observations that only 13 genes were subject to regulation by all three spinach extracts, and only 23 of the 586 CWPS-regulated genes were also regulated by both the spinach and lettuce extracts.

**FIGURE 3:**
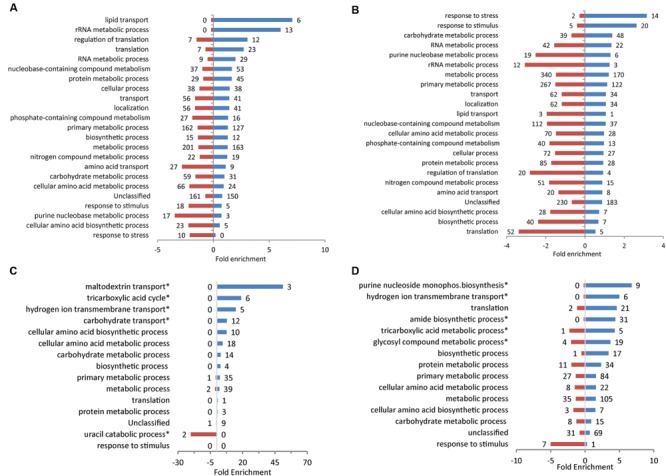
**GO term enrichment for response to plant extracts.** GO terms for *E. coli* O157:H7 (Sakai) genes that were significantly differentially expressed following growth in spinach leaf lysates **(A)**, spinach root exudates **(B)**, CWPS extracts from spinach **(C)** or lettuce **(D)**, relative to their respective controls. Data was obtained from the Gene Ontology Consortium website. Significantly enriched Biological Processes are shown for induced (blue) and repressed genes (red), using GO-Slim and selected GO-complete categories (indicated by ‘^∗^’; full list in Supplementary Table S2). The numbers of individual genes are adjacent to each bar on the charts.

To determine which groups of genes were affected by exposure to the plant extracts, analysis was performed for genes annotated with GO terms. GO-term enrichment enabled identification of over- or under-represented groups of genes that were differentially expressed by each treatment. Analysis of significantly enriched (*p* < 0.05) ‘Biological Processes’ were performed on a broad-scale level using GO-Slim terms, and supplemented with GO-Complete for a more a detailed breakdown of smaller classes of genes (**Figure 3**; Supplementary Table S2). When all of the plant extract treatments are considered together, Metabolic Processes is the category with the largest number (41–510) of affected genes, although there are clear differences between treatments in the ratio and number of up and down-regulated genes, and for different specific metabolic classes (**Figure 3**). Exposure to spinach leaf lysate resulted in differential expression of 364 genes in metabolic processes (163 induced, 201 repressed). The highest level of enrichment was for induced genes associated with lipid transport (six induced; sevenfold enriched) and there was significant positive enrichment for translation-related processes (translation, rRNA metabolism, regulation of translation) and protein metabolism (48, and 45 induced, respectively). Genes involved in primary metabolism were subject to a high degree of control with 127 genes induced and 162 repressed. Following exposure to spinach root exudates, a large number of genes associated with metabolic processes were down-regulated genes (340), with half as many (170) induced. In general, more down-regulated genes were enriched for the different GO term categories compared to those induced (67 vs. 33% in **Figure 3B**). The highest level of enrichment for repressed genes was seen for those associated with translation-related processes (translation, rRNA metabolism, regulation of translation), of which 84 were down-regulated and just 12 induced; this pattern is the reverse of that seen above for spinach leaf lysate. The highest enrichment for induced genes (14) was in response to stress. These expression effects thus suggest that exposure to spinach root exudates caused increased stress combined with reduced translation capacity.

Upon exposure to spinach CWPS, the enriched gene GO terms categories were almost entirely represented by induced genes (159/165; **Figure 3C**). The largest group was in metabolic processes (39 induced, two repressed) and the highest level of enrichment was seen for a small class associated with maltodextrin transport (three genes induced; 51-fold enrichment). Exposure to lettuce CWPS resulted in a similar pattern of enrichment as for spinach CWPS, with the majority of the groups associated with different categories of metabolism, e.g., 105 induced, 35 repressed in metabolic processes (**Figure 3D**). Furthermore, most genes in each category were induced (as above for spinach CWPS), with only one category (response to stimulus) having a greater level of enrichment for repressed genes (seven repressed; fivefold enrichment).

In general, exposure to the different plant extracts generated distinct patterns of GO term enrichment (**Figure 3**), which was also distinct from that for growth at 18°C (**Supplementary Figure S1**). However, some although some commonalities in enrichment occurred for the more specific categories. Furthermore, a large proportion of genes for each treatment type fell outside the GO annotations that are not considered by the enrichment analysis. Therefore, to examine the regulatory response in more detail, individual genes, or groups of related genes, are compared for each treatment type in more detail below.

### Metabolism

**FIGURE 4:**
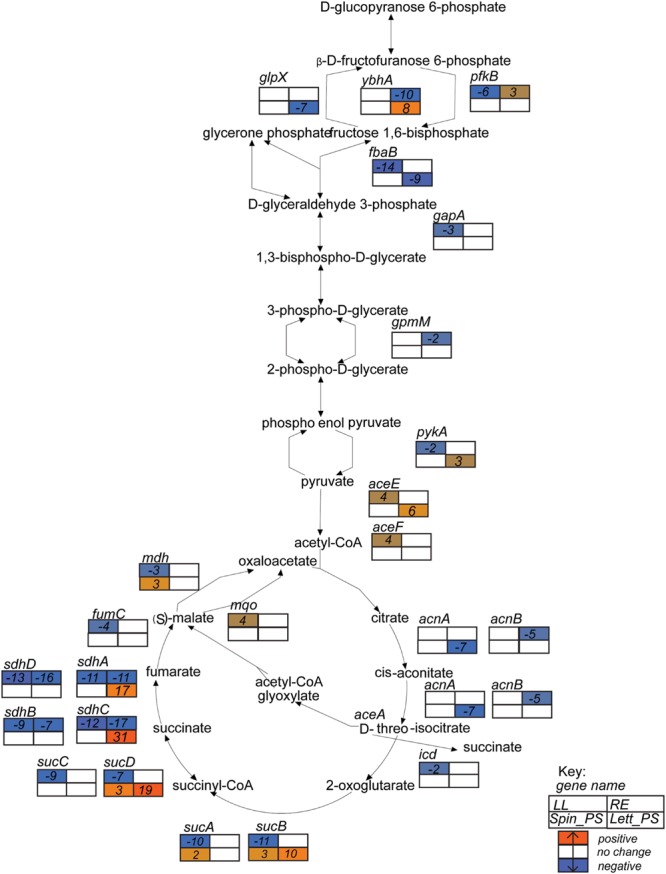
**Glycolysis superpathway gene expression profiles.** Expression data for *E. coli* O157:H7 (Sakai) in response to different plant extracts was overlaid onto the metabolic pathway in EcoCyc (Keseler et al., 2013) to generate a color scale of expression from orange for induction to blue for repression and white for no change < ± twofold. Expression is provided for relevant genes in the pathways that were changed in at least one of the four plant conditions. Gene names are in italics and placed adjacent or close to their relevant substrates. The data for all four conditions are arranged in a grid, ordered as indicated in the Key: LL, leaf lysates and RE, root exudates for spinach; Spin_PS, spinach CWPSs; Lett_PS, lettuce CWPSs.

As indicated above, metabolism encompasses the largest number of differentially expressed genes for all of the extracts tested, although there were major differences between extracts. Components of glycolysis and the Krebs cycle that were induced on exposure to lettuce CWPS included enzymes required for the conversion of oxoglutarate to succinyl-CoA (*sucAB*, 17- and 20-fold), succinate (*sucCD*, 8- and 29-fold) and fumarate (*sdhABCD*, 11–38-fold), and for malate oxidation (*mqo*, sixfold; **Figure 4**). This was coupled with a 20-fold induction of the gene encoding the DctA symporter, required for aerobic uptake of C_4_-dicarboxylates such as succinate (Davies et al., 1999; **Supplementary Figure S2**). The main gene associated with central metabolism that was induced on exposure to root exudates was acetyl-CoA synthetase (*acs*, fivefold). The gene (*pdhR*) encoding the pyruvate-dehydrogenase complex regulator (PdhR, an autoregulatory repressor responding to pyruvate) was induced on exposure to both spinach leaf lysate (fourfold) and lettuce CWPS (ninefold), as were the three genes in the PdhR-controlled *aceEF-lpd* operon (Supplementary Table S1). However, induction of *lpdA* was higher than that of *aceEF* in lettuce CWPS (23- cf. 4- to 6-fold) which reflects *lpdA* expression from an independent promoter and the involvement of lipoamide dehydrogenase (E3) component in both the pyruvate dehydrogenase and 2-oxoglutarate dehydrogenase multienzyme complexes (Cunningham et al., 1998). In contrast, *pdhR* was 15-fold repressed in root exudates. These findings indicate low cellular pyruvate levels upon exposure to root exudates, suggestive of low carbon source availability (see below).

Exposure to spinach root exudates or lettuce CWPS resulted in up-regulation of the methylgalactose uptake operon (*mglABC*) by 6–8-fold, but this was subject to eightfold repression by spinach leaf lysate (Supplementary Table S1). Lactose utilization genes (*lacZY*) were induced (ninefold) only in lettuce CWPS extracts, while genes required for utilization of sorbitol (*srlAEBDMRQD*) were 10-fold induced in root exudates but fourfold repressed in leaf extract. Genes for xylose metabolism (*xylAB*) were also induced, fivefold, in root exudates. Fatty acid degradation (*fadABDEHIJKL*) genes were strongly induced (56-fold) by spinach root exudates while fatty acid synthesis genes (*fabHDG-acpP-fadF,fadABIZ*) were twofold repressed. This reciprocal regulation of the fatty acid systems is likely explained, in part, by the threefold repression of *fadR*, encoding the fatty-acid responsive *fad* gene repressor, and the sixfold induction of *fabR* specifying a repressor of *fab* genes. The regulatory response observed suggests enhanced availability of fatty acids in the root exudates. The reverse response was seen in lettuce CWPS: a threefold repression of the *fad* genes and a fourfold induction for the *fad* genes, indicating low fatty acid availability under this condition. Genes involved in purine and pyrimidine biosynthesis (*purABCDEFHKLMNTU, carAB, pyrDFI*) were the most strongly induced (average of 36-fold) genes on exposure to lettuce CWPS, but were 18- and 4-fold repressed by spinach root exudates and leaf extract. This indicates availability of nucleotide precursors in the root and leaf samples, but not in lettuce CWPS. Similarly for arginine, since carbamyl phosphate is regulated jointly by arginine and pyrimidines through transcriptional repression of carbamoyl phosphate synthase *carAB* (Caldara et al., 2006), which was evident in spinach leaf lysates and root exudates coupled with repression of *arg* and *art* genes (average 13-fold reduction), whereas in contrast arginine biosynthesis genes were induced in lettuce CWPS by 3- to 48-fold for *argC,E,G,S* and *artIJ*.

Exposure to the plant extracts induced changed in global regulators that play a functional role in control of growth. Expression of the gene encoding the factor-for-inversion stimulation protein (*fis*) was induced on exposure to spinach leaf lysates (threefold) and repressed (28-fold) in spinach root exudates (Supplementary Table S1). CsrA, a glycolysis activator and a gluconeogenesis repressor was induced fivefold in the presence of spinach root exudates. In addition, genes encoding the RNA polymerase subunits for the core enzyme, α, β, β′, and ω and sigma subunit 70 were all induced in leaf lysates (3- to 15-fold), whereas only the alternative sigma subunits for sigma E, sigma H and sigma S were marginally induced in spinach root exudates (twofold).

Iron acquisition is often linked to growth and division (Kohler and Dobrindt, 2011), and the extracts induced markedly different responses in associated systems. The *ent* genes encoding synthesis of the siderophore enterobactin were upregulated on exposure to spinach root exudates (2–11-fold), but not the leaf lysates (Supplementary Table S1), which might be partly explained by the ∼threefold reduced expression of the global iron-responsive repressor, Fur, in the root exudates. Similarly, expression of the haem-transporter (*chu*) genes were induced in root exudates (3–20-fold compared), but not in spinach leaf lysates. These results suggest iron restriction is imposed by the root exudates, but not by the leaf extract. In contrast, the ferrous-iron-transport system (*feoABC*) genes were repressed for *E. coli* O157:H7 (Sakai) in spinach root exudates (∼3-fold). The iron-storage proteins were induced in two of the extracts: *ftnA* (fivefold) in lettuce CWPS; and *ftnA* and *bfr* (both ∼threefold) in spinach leaf lysates. The IscR-regulated gene cluster (*iscRSUA-hscBA-fdx-iscX*), associated with Fe–S cluster assembly was induced (average of 8.2-fold) in lettuce CWPS, but was fourfold repressed in spinach root extract.

### Stress Responses

The genes most strongly affected by exposure to leaf and root extracts were those associated with response to various stresses. The *asr* gene (acid-shock inducible periplasmic protein) was the most strongly induced gene on exposure to spinach leaf lysates (240-fold) and root exudates (637-fold), but not significantly affected in either of the CWPS extracts. Regulators and functional enzymes involved in glutamate-dependent acid resistance included the acid fitness island regulators, *gadWX* (Tramonti et al., 2008), repressed sixfold in spinach leaf lysates and 15- and 26-fold, respectively in lettuce CWPS, and but induced eightfold in spinach root exudates. Induction of *gadAB* and *gadC* encoding the glutamate decarboxylase and glutamate:gamma-aminobutyric acid antiporter occurred in root exudates (2–8-fold) in contrast to *gadA* repression in spinach leaf lysates or lettuce CWPS (12- or 21-fold, respectively), which supports regulatory control and response of the glutamate-dependent acid resistance system. However, it was notable that *gadE*, a central activator of the response (Ma et al., 2003), was not differentially affected on response to root exudates.

Many of the genes encoding the cold shock proteins (*cspA-I*) were subject to regulatory change by the plant extracts (Supplementary Table S1). This was particularly clear for the spinach leaf lysates and root exudates where there appeared to be a reciprocal response: *cspA* and *cspF-I* were 12-fold induced in leaf lysate, but fivefold repressed in root lysate; whereas *cspD* was eightfold repressed or 12-fold induced, respectively. Genes encoding the universal stress proteins (*uspB,C,D,E,F*, and *G*) were induced in root exudates (average of 10-fold), although three of these genes (*uspB,D*, and *F*) were 12–29-fold repressed in lettuce CWPS. *spoT*, associated with the stringent response, was moderately induced in response to spinach leaf lysates (twofold), but repressed sixfold in root exudates. Stress-response genes, e.g., *spoT* and cold shock genes play a functional role in response to metabolic-related changes and may reflect translational stalling (discussed below).

### Motility and Adherence

**FIGURE 5:**
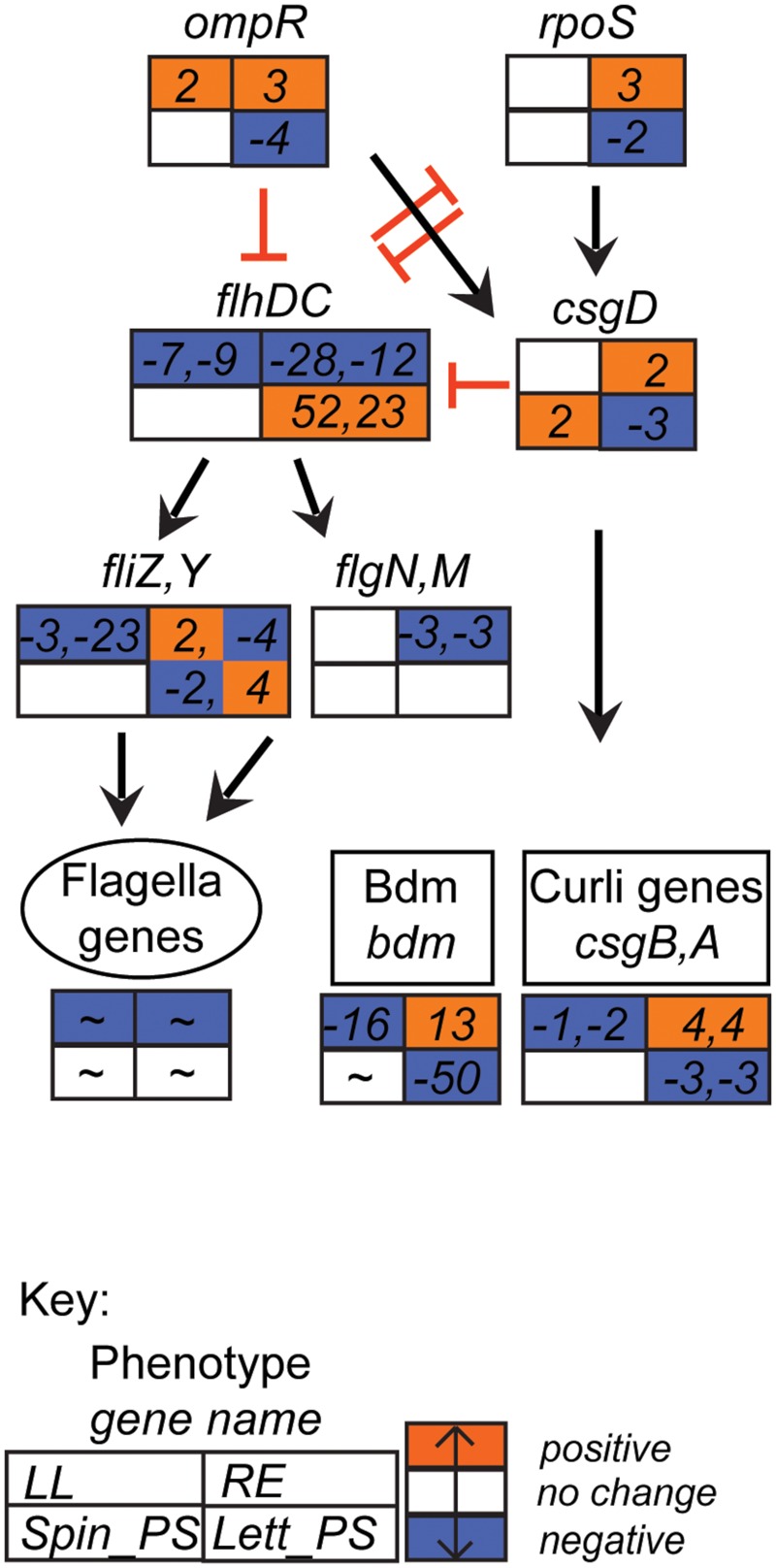
**Flagella-curli regulatory network gene expression profiles.** Expression data for selected *E. coli* O157:H7 (Sakai) genes in response to different plant extracts was overlaid onto the network to generate a color code of expression: orange for induction, blue for repression, and white for no change < ± twofold. Expression is provided for selected genes that were affected in at least one of the four plant extracts. Gene names are in italics; genes associated with a phenotype are grouped together; an overall approximate change (indicated by ‘∼’) is provided for flagella genes (data in Supplementary Table S2B). The data for all four conditions is arranged in a grid, ordered as indicated in the Key: LL, leaf lysates and RE, root exudates for spinach; Spin_PS, spinach CWPSs; Lett_PS, lettuce CWPSs. Regulatory connections, both direct and indirect (Pesavento et al., 2008; Pesavento and Hengge, 2012; Guttenplan and Kearns, 2013), with either positive (black arrow) or inhibitory (red bar) effects are shown.

Gene associated with motility and biofilm formation are often associated with successful colonization of plants (Cooley et al., 2003; Van Houdt and Michiels, 2010). Both groups were strongly repressed in the baseline condition of growth in minimal medium at 18°C compared to 37°C, as indicated above. However, upon exposure to spinach whole-leaf lysates or root exudates, the genes encoding the master motility regulator FlhDC were repressed 7–28-fold, but were induced 23- and 52-fold (respectively) on exposure to lettuce CWPS (**Figure 5**). In lettuce CWPS, this increase in motility-gene regulator expression was coupled with repression of the biofilm-related gene, ECs2085 (*bdm*; 50–55-fold repressed) encoding the biofilm-dependent modulation protein, and a modest effect on the genes encoding curli fibers (*csgA,B*: both threefold). In contrast, curli genes were induced on exposure to spinach root exudates (also by threefold; Supplementary Table S1), indicative of a switch between sessility vs. motility. Some of the genes encoding fimbriae were induced, but only to moderate levels. For example, multiple signals for *loc2* were induced in response to root exudates, including ECs0142 (*yadM*, a putative structural subunit) and *yadK* (also a structural subunit), by 2- and 3-fold, respectively.

### Hypothetical Genes

Genes annotated as hypothetical accounted for a large number of differentially expressed genes for all four treatments: 432, 603, 7, and 119 genes for spinach leaf lysates, root exudates, spinach CWPS and lettuce CWPS, respectively (Supplementary Table S1). They also accounted for high levels of differential expression: e.g., in spinach leaf lysates two hypothetical genes (b3238, b1722) were ranked as #2 and 3 for level of induction, at ∼50-fold. Probes corresponding to Z5022 and ECs4474 were induced 270- to 300-fold in spinach root exudates, but repressed in spinach leaf lysates and lettuce CWPS (3–92-fold). Some of these genes are unique to the O157:H7 serotype (**Table 1**) and not present in the closely related O157:H7 isolate EDL933. Four of these were differentially expressed in spinach leaf lysates or lettuce CWPSs: ECs1375, ECs2713, ECs4970 and ECs4976, ranging between threefold repressed and sevenfold induced. It is possible that some of these genes play a distinct role in plant colonization that has not yet been investigated.

**Table 1:** Expression of selected genes encoding for hypothetical proteins of *E. coli* O157:H7 (Sakai).

Accession #	MG1655	EDL933	Top BLASTn hit (nt identity %)	Gene expression data (fold-change)			
				Spinach LL	Spinach RE	Spinach CWPS	Lettuce CWPS
ECs0317	✓	✓	Membrane protein, *Escherichia albertii* KF1 (97)	11.85	4.81	NS	NS
ECs0845	x	✓	Bacteriophage tail protein HUN/2013, TL-2011C, Min27 (98)	NS	5.00	NS	NS
ECs0988	✓	✓	*yeaO, Shigella flexneri* (99)	2.49	-18.16	NS	12.06
ECs1254	✓	✓	Putative enzyme, *Shigella dysenteriae* (90)	NS	NS	-4.92	-8.98
ECs1335	x	x	*Shigella flexneri* plasmid pSFxv_1 (90)	3.42	NS	NS	4.53
ECs1375	x	x	No highly similar BLAST hits outside *Escherichia coli* (n/a)	2.87	NS	NS	NS
ECs1653	x	✓	*Citrobacter freundii* CFNIH1 (95)	-15.29	9.20	NS	-13.23
ECs1654	x	✓	*Citrobacter freundii* CFNIH1 (94)	-32.08	8.79	NS	-71.95
ECs1655	✓	✓	*Citrobacter freundii* CFNIH1 (94)	-18.13	8.85	NS	-8.21
ECs2304	✓	✓	*Shigella dysenteriae* Sd197 (99)	12.97	20.21	NS	-3.27
ECs2473	x	✓	*Escherichia albertii* KF1 (81)	NS	NS	NS	11.65
ECs2489	✓	✓	*yeaD, Shigella dysenteriae* Sd197 (99)	-3.60	-2.96	2.50	3.30
ECs2713	x	x	Putative cytochrome, *Shigella boydii* (97)	-2.89	NS	NS	-4.18
ECs2940	x	x	Bacteriophage tail fiber protein, *Escherichia albertii* KF1 (99)	NS	4.72	NS	NS
ECs3238	x	✓	No highly similar BLAST hits outside *Escherichia coli* (n/a)	NS	11.80	NS	-2.29
ECs3521	✓	✓	FAD dependent oxidoreductase *csiD, Shigella boydii* (97)	-12.88	NS	NS	-8.91
ECs3750	✓	✓	Conserved hypothetical protein, *Shigella boydii* Sb227 (99)	NS	34.86	NS	-12.47
ECs4115	✓	✓	*aaeX, Shigella flexneri* 2002017 (99)	24.15	2.86	NS	NS
ECs4474	✓	✓	*yibI, Shigella flexneri* Shi06HN006 (99)	-3.10	267.91	NS	-92.50
ECs4491	✓	✓	M23 peptidase domain protein, *Shigella boydii* CDC 308394 (98)	NS	-19.12	NS	10.08
ECs4970	x	x	Galactidol-1-phosphatol dehydrogenase, *Citrobacter rodentium* (94)	NS	NS	NS	4.87
ECs4976	x	x	Galactidol-1-phosphatol dehydrogenase, *Citrobacter rodentium* (92)	NS	NS	NS	7.13
ECs5165	✓	✓	Biofilm stress and motility protein A, *Shigella flexneri* Shi06HN006 (99)	-6.01	60.15	NS	-7.03

*Homologs in *E. coli* K-12 strain MG1655 and O157:H7 isolate EDL933 are indicated by ‘✓’ or ‘x’ for presence/absence and the top BLASTn hit provided with the percentage of nucleotide identity. The gene expression data from microarray analysis is provided as fold change (relative to the appropriate control; NS is not significant), for each of the four plant extracts: spinach leaf lysates (LL) and root exudates (RE); and cell wall polysaccharides (CWPSs) from spinach and lettuce.*

### Colonization Potential is a Reflection of Adaptive Gene Expression

**FIGURE 6:**
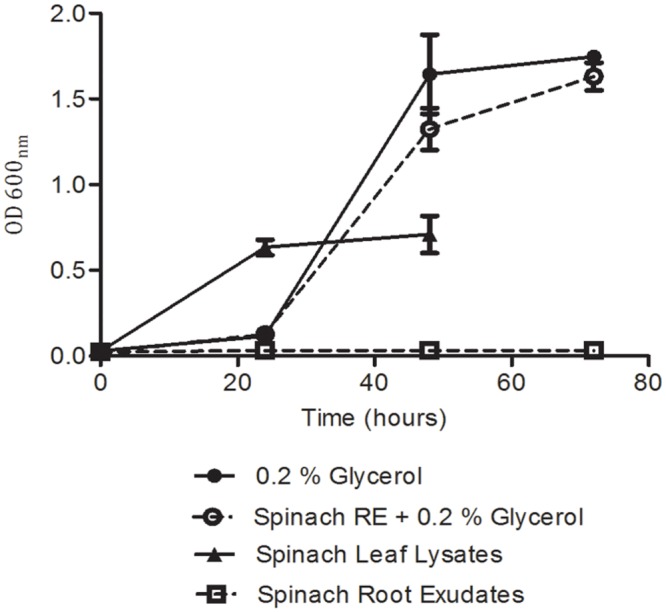
**Growth analysis for *E. coli* O157:H7 (Sakai) in spinach plant extracts.** Growth was quantified from cell density of bacteria inoculated into M9 medium supplemented with plant extracts and/or glycerol. Measurements were discontinued for the leaf lysate condition after 48 h, as growth was complete. Data represents the average of nine replicates and was analyzed by one-way ANOVA at selected time points.

To determine the extent to which the global-gene-expression changes reflect the colonization potential of the bacteria in different plant tissue extracts, the ability of the plant tissue extracts to support *in vitro* growth was assessed. For these assays, minimal M9 medium was used as a basal medium (without carbon source) supplemented with spinach leaf lysate or root exudates (normalized on the basis of protein content), or with 0.2% glycerol as a ‘no-plant’ control. Bacterial growth could not be assessed in medium containing the (insoluble) CWPS extract and as such, is not considered here. *E. coli* O157:H7 (Sakai) grew well in medium supplemented with spinach leaf lysate at 18°C, reaching an OD_600_ of 0.7 at 48 h, which was just-under 50% of that (1.7) achieved in M9 medium plus 0.2% glycerol (**Figure 6**). In addition, growth with the leaf lysate exhibited a very short lag phase, unlike that with glycerol where a ∼24 h delay in rapid growth was observed. This suggests that the bacteria acclimatized more rapidly to the medium with leaf extract than that with glycerol. In contrast, no growth was evident with spinach root exudate suggesting that carbon was at least one of the limiting energy sources. Indeed, when the root exudate and glycerol were used in combination strong growth was obtained that was similar to that with glycerol alone, suggesting that the weak growth in spinach root exudates was not due to the presence of factors that supress growth (**Figure 6**). No significant difference was found between the growth of *E. coli* O157:H7 (Sakai) in the glycerol only media compared to the glycerol plus root exudates media. To test whether the root exudate was deficient in suitable carbon sources, the composition of mono- and disaccharides in the extracts was examined by HPLC. The analysis showed ∼200-fold less glucose, fructose, and sucrose in the root exudate compared to the leaf lysate, supporting the suggestion that the root exudate provides limited levels of carbohydrate (**Table 2**). Although root exudates were collected from plants grown under aseptic hydroponics conditions, and germinated from surface-sterilized seeds, it was apparent that there were native bacteria associated with the spinach plants. Cultivable bacteria were tentatively identified as *Pseudomonas azotoformans* (with 99.90% nt identity) and *Pantoea agglomerans* (99.95% nt identity). In our hands, the contaminating bacteria were repeatedly associated with spinach grown under these conditions indicating that they were seed-borne.

**Table 2:** Mono- and disaccharide content in the plant extracts, as measured by HPLC.

Monosaccharide (μg/mg dry weight)	Spinach leaf lysate	Spinach root exudate	Spinach CWPS	Lettuce CWPS
Arabinose	502	0	18.1	3.59
Rhamnose	0	0	3.67	0.59
Glucose	289	2.80	87.1	22.1
Fructose	231	2.26	51.3	23.7
Sucrose	73.2	0.78	0.48	0.27
**Total**	*1090*	*5.84*	*161*	*50.3*

### *E. coli* O157:H7 (Sakai) Colonization Potential of Roots and Leaves of Spinach, Lettuce, and Vining Pea Plants

To examine the longer-term outcome of bacterial adaptation to the plant environment, the colonization potential of *E. coli* O157:H7 (Sakai) was determined on living plants over 10 days. Here, ‘colonization potential’ is defined as a measure of the ability of the bacteria to survive and/or grow. Colonization potential was tested on the leaves and the roots of both spinach and lettuce, as above, and also on vining green pea (*Pisum sativum*), which is eaten raw as pea shoots, and wild prickly lettuce (*Lactuca serriola*), an ancestral relation of lettuce. In all cases, the whole *E. coli* O157:H7 population was enumerated with no attempt made to distinguish epiphytes from endophytes.

**FIGURE 7:**
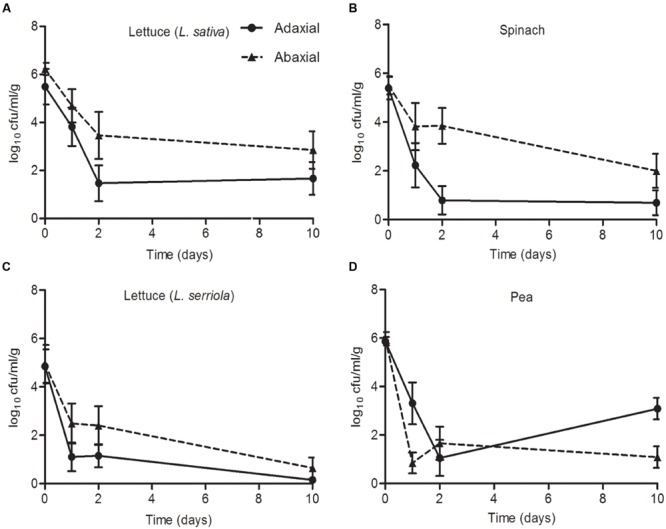
***Escherichia coli* O157:H7 (Sakai) on leaves.** The numbers of *E. coli* O157:H7 (Sakai) recovered from the leaves of roundhead lettuce [*L. sativa*; **A**), spinach (*Spinacia oleracea*; **B**), wild prickly lettuce (*Lactuca serriola*; **C**), and vining green pea (*Pisum sativum*; **D**], following inoculation of the abaxial (black triangles) or adaxial (black circles) surfaces with a starting inoculum of 2 × 10^6^ CFU (Log_10_ 6.3). The average and the standard error of the mean for six replicates for each of the time points is expressed as the number of CFU recovered per gram of fresh tissue (Log_10_). Data is generated from nine replicate samples and was analyzed by one-way ANOVA with the Tukey multiple comparison test.

An *E. coli* O157:H7 (Sakai) inoculum of 6.3 log_10_ CFU was applied to the adaxial (upper) and abaxial (lower) surface of the leaves of four different plant species and the bacteria enumerated over 10 days. There was a decrease in bacterial numbers compared to the starting inoculum for all four species, on both leaf surfaces. However, in each case, a higher average number of *E. coli* O157:H7 (Sakai) was recovered from the abaxial than adaxial surface after 10 days (**Figure 7**), although the difference was not significant at the 95% confidence level. The average number of *E. coli* O157:H7 (Sakai) on both leaf surfaces of both species of lettuce (*L. sativa* and *L. serriola*) decreased over the time tested, although the numbers recovered at d10 were significantly different: 1.66/2.84 (adaxial/abaxial) log_10_ CFU for *L. sativa* and at the limit of detection (0.15/0.63 log_10_ CFU, adaxial/abaxial) for *L. serriola* (*p* < 0.05), with bacteria only recovered from 22% of the samples for *L. serriola* for this time point. The number of *E. coli* O157:H7 (Sakai) on spinach also decreased from the starting inoculum and although higher counts were obtained from the abaxial side of the leaf at d2, by d10 they had reached similar levels, stabilizing at 0.69 adaxial and 1.99 abaxial log_10_ CFU. Pea was the only plant where the numbers increased between d2 and d10, from 1.05 to 3.08 log_10_ CFU (abaxial). By d10, significantly higher numbers were recovered found pea than *L. serriola* (adaxial, *p* < 0.01; abaxial, *p* < 0.05).

**FIGURE 8:**
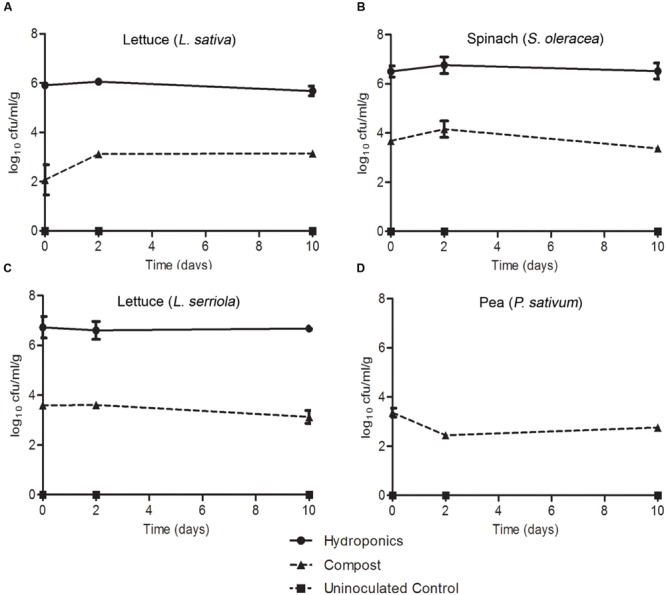
***Escherichia coli* O157:H7 (Sakai) on roots.** The numbers of *E. coli* O157:H7 (Sakai) recovered from the roots of roundhead lettuce (*L. sativa*; **A**), spinach (*S. oleracea*; **B**), wild prickly lettuce (*L. serriola*; **C**), and vining green pea (*P. sativum*; **D**), following inoculation of the compost (triangles) or hydroponics liquid media (circles). *P. sativum* was not grown under hydroponics conditions. Bacteria were not recovered from the hydroponic media-only control (squares). The average and the standard error of the mean for each of the time points is expressed as the number recovered per gram of fresh tissue (Log_10_). Data is generated from nine replicate samples and was analyzed by one-way ANOVA with the Tukey multiple comparison test.

Colonization of roots was compared for plants grown in compost or hydroponics medium, to partly account for any potential effect from native compost-associated microbiota. Inoculation of compost-grown plants was achieved by partially immersing the plant pots in a bacterial suspension at 7.3 log_10_ CFU/ml, which resulted in the recovery of between 2.0 and 4.0 log_10_ CFU/g *E. coli* O157:H7 (Sakai) from the roots at the initial time point (1 h post inoculation; **Figure 8**). Despite some variation between plant species, the bacterial populations remained relatively stable and did not decrease as observed on leaves. *E. coli* O157:H7 (Sakai) recovered from *P. sativum* roots decreased marginally at day 2 but increased again by d10. Highest recovery at d10 occurred from spinach, followed by *L. serriola, L. sativa* and pea (3.4, 3.35, 2.76, and 2.24 log_10_ CFU, respectively). For the colonization potential of *E. coli* O157:H7 (Sakai) on roots of plants grown under hydroponics (liquid) conditions, the inoculum (7 log_10_ CFU/ml) was introduced into the medium adjacent to the roots. The number of *E. coli* O157:H7 (Sakai) recovered at the first time point was ∼2 orders of magnitude higher than that for compost-grown plants. The levels of *E. coli* O157:H7 (Sakai) recovered after 10 days were at least as high, or higher, than the initial inoculum (**Figure 2**). Greater recovery of bacteria occurred from *L. serriola* and spinach than *L. sativa* at d10 (7.04, 6.36, and 5.88 log_10_ CFU, respectively). No proliferation of *E. coli* O157:H7 (Sakai) occurred in the hydroponics medium in the absence of plant roots, with the population at 4.46 log_10_ CFU at d10, significantly different to *E. coli* O157:H7 (Sakai) from the three plants (*p* < 0.001). In our hands, it was not possible to remove surface-associated fungi from *P. sativum* seeds sufficiently well to allow its growth under aseptic hydroponics conditions; therefore, this combination was not tested. These experiments demonstrate that *E. coli* O157:H7 (Sakai) was able to either stabilize or increase its population on leaf and root, but that there were plant, tissue and growth media specific differences that affected colonization potential.

## Discussion

The aim of the experiments reported here was to examine adaptation to and colonization of a key crop-plant-associated pathogen (*E. coli* O157:H7 Sakai) to the leaves and roots of four distinct leafy vegetables. Examination of the initial expression response of the pathogen upon exposure to the plant allowed for assessment of the physiological changes that facilitate adaptation to the plant niche. *E. coli* O157:H7 (Sakai) was found to survive on the leaves of all four plants (two lettuce species, spinach and pea) over a 10-days period, although the numbers of cultivable bacterial declined from a high starting inoculum over the first 1–2 days. Differences in degree of survival and the effect of time were observed suggesting that the bacteria experienced distinct leaf environments during their colonization of each of the four plants tested, which affected their recovery. Survival of *E. coli* O157 was superior in the root environment, with little decline in bacterial number observed over a 10-days period. However, again there were differences in bacterial recovery between the four plant species indicative of a species distinct impact on bacterial adaptation and survival.

The physiological response of *E. coli* O157 in response to plant extracts was examined in relation to persistence of *E. coli* O157 on leaves and roots. To facilitate this, extracts from spinach leaves and root exudates were used. Plant CWPS extracts from lettuce and spinach were included in an attempt to identify species-specific differences in response to plant factors. Extracts, rather than the live plant, were used to ensure sufficient bacterial recovery for expression analysis, to eliminate plant defense effects and to strictly control expression conditions to achieve good reproducibility. Such an approach has been used successfully by others previously (Kyle et al., 2010). However, use of extracts removes the plant host-dependent dynamic that could affect the bacterial response in comparison to the situation on live plants. The time of exposure was limited to just 1 h, which represents the period of initial adaptation. The four plant extracts induced marked differences in the transcript profiles for *E. coli* O157:H7 (Sakai) during the short (1 h) exposure at 18°C, reflective of adaptation toward active metabolism and growth. The spinach-leaf extract was shown to support growth of *E. coli* O157:H7 (Sakai) and although root exudate failed to enable such growth (due to an apparent lack of carbon source), it did not significantly inhibit growth when a suitable carbohydrate was provided. These observations suggest that the bacteria remain metabolically active and capable of mounting a regulatory response to their new environment during the 1 h exposure to the plant extracts.

Temperature is a major factor in differential gene expression (Phadtare and Inouye, 2008) likely to have influenced data obtained in many previous global-expression studies on bacterial colonization. Thus, the conditions employed here were controlled to ensure that the only change influencing *E. coli* O157:H7 (Sakai) gene expression was the introduction of plant extract to the culture medium. Indeed, this approach was vindicated by large-scale changes in gene expression (more than 20% of the genome) induced by growth at 18°C (plant-relevant temperature) instead of 37°C (mammal-relevant temperature). Since a cold shock from 37 to 14°C has been shown to result in induction of *fli* and *flg* genes (Phadtare and Inouye, 2004), the observed repression of these genes at 18°C compared to 37°C supports the lack of any cold shock imposed on *E. coli* O157:H7 (Sakai) under the conditions tested here. Furthermore, repression of genes associated with the type 3 secretion system (T3SS), in particular substantial down-regulation of the master regulator *ler*, support previous reported data on thermoregulatory control of T3SS in pathogenic *E. coli* at sub-mammalian temperatures (Umanski et al., 2002).

Other laboratories have investigated various aspects of the transcriptional response of *E. coli* to fresh produce (Kyle et al., 2010; Fink et al., 2012; Hou et al., 2012, 2013; Landstorfer et al., 2014; Linden et al., 2016) and alternative approaches have investigated genes required for plant-associated bacteria to colonize plant hosts (e.g., Silby et al., 2009). One of the most directly comparable studies examined early expression profiles of *E. coli* O157:H7 strain EDL933 to lettuce leaf lysates (Kyle et al., 2010), to mimic the bacterial response to damaged plant tissue. There are some parallels with these studies, such as up-regulation of genes involved in transport of metabolites (Kyle et al., 2010), but important specific differences occurred that are likely to have arisen from differences in the experimental approach.

In general, the transcriptome analysis paints a picture of *E. coli* O157:H7 (Sakai) undergoing a transition toward attempts at active growth, captured at different stages for the different extracts. Each of the plant extracts induced distinct transcriptional profiles for *E. coli* O157:H7 (Sakai), although metabolism was a common category. Growth phase transitions are known to induce significant changes in metabolite gene expression and production (Jozefczuk et al., 2010), which was reflected here by expression of genes involved in glycolysis and the Krebs cycle, e.g., induction of the genes for succinate and fumarate conversion in the presence of CWPS.

Several pieces of evidence show that *E. coli* O157:H7 (Sakai) was in a lag phase and in transition to growth following a 1 h exposure to spinach leaf lysates. The factor-for-inversion stimulation protein (FIS) was one of the most strongly induced global regulators in spinach leaf lysates. FIS is DNA binding protein that modulates chromosome dynamics and is highly induced during lag phase as the cells are preparing to divide (Schneider et al., 1997). Induction of MQO in leaf lysates, and repression of malate dehydrogenase (*mdh*), supports the idea that MQO can sustain low levels of TCA-cycle activity independent of MDH activity (van der Rest et al., 2000), and may also indicate that *E. coli* O157:H7 was undergoing transition to exponential phase. Induction of the pyruvate dehydrogenase system (*phdR,ace,aceF,lpd*) indicated the presence of pyruvate on exposure to both spinach leaf lysates and lettuce CWPS, since the operon is de-repressed in the presence of the carbohydrate (Quail et al., 1994). The pyruvate dehydrogenase complex is central to metabolism where PdhR is a master regulator of the genes involved for the transfer of pyruvate, the final product of glycolysis, into the Krebs/TCA cycle (Ogasawara et al., 2007).

The experimental set-up to investigate the response to plant extracts was designed not to incur a temperature shift, yet *cspA* and *cspG* were highly induced on exposure to spinach leaf lysates and lettuce CWPS. Cold shock proteins function as RNA chaperones, either re-folding misfolded transcripts or presenting them for degradation by RNases (Yamanaka et al., 1998), and are induced following translational stalling, e.g., on a shift to low temperatures or other ‘stress-response’ conditions. CspA and CspG RNA chaperones are highly expressed during antibiotic-driven translation inhibition (Etchegaray and Inouye, 1999) and their induction from spinach leaf lysates and lettuce CWPS coupled with the induction of *spoT*, a marker of the stringent response, supports the idea of a pause in translation during adaptation to the new environment. This may also explain induction of two *E. coli* O157:H7 (Sakai) cold shock genes on exposure to lettuce leaves of living plants (Linden et al., 2016). In contrast, *E. coli* K-12 *csp* genes were shown to be repressed on exposure to lettuce leaves elsewhere (Fink et al., 2012), although differences in the experimental set-up and baseline comparison may explain the observations.

Expression of *cspD* is indicative of nutrient stress (Yamanaka et al., 1998), and it was repressed in leaf lysates and lettuce CWPS extracts but induced following exposure to spinach root exudates, supporting the inability of *E. coli* O157:H7 (Sakai) to grow in this extract (**Figure 6**). This was further supported by induction of the *usp* family of genes, related to a variety of environmental assaults including DNA damage, oxidative stress and iron limitation (Nachin et al., 2005). Induction of the glutamate acid stress response system in root exudates was indicative of a response to acidic conditions in root exudates. The opposite response in spinach leafy lysates and lettuce CWPS indicated the presence of polyamines (spermidine and putrescine) that are known to repress the glutamate decarboxylase dependent acid response in *E. coli* (Chattopadhyay and Tabor, 2013).

There was evidence for catabolite control in response to CWPS and root exudates, from induction of high-affinity transport systems for malate and galactose normally seen under glucose-limiting conditions (Franchini and Egli, 2006) and induction of *lacZY*, in lettuce CWPS. There was evidence for degradation of carbohydrates (xylose and sorbitol) in spinach root exudates. A similar scenario of glucose-limitation was reported for *E. coli* O157:H7 (EDL933) in response to lettuce leaf lysates, e.g., with high levels of induction of genes for malate and sorbose uptake and metabolism (Kyle et al., 2010). Further evidence for use of alternative metabolites was from induction of acetyl-CoA synthetase (*acs*), which converts acetate to acetyl-CoA and is central to several metabolic pathways including the TCA cycle (Pietrocola et al., 2015). Changes in metabolic flux were also indicated by the presence of CsrA (and CsrD; Romeo, 1998). Fatty acid degradation (*fad* genes) and fatty acid synthesis (*fab* genes) is tightly balanced in the cell and co-regulated by FadR, a master regulator that represses *fad* genes and activates *fab* genes (My et al., 2015). In spinach root exudates the balance was tipped strongly toward fatty acid degradation, while the opposite occurred in spinach leaf lysates, indicative of membrane biogenesis required for active growth. Fatty acid degradation was also observed for colonization of *Pseudomonas fluorescens* (isolate SWB25) on sugar beet seedlings (Silby et al., 2009).

Iron scavenging is linked to growth and can also be associated with successful colonization of hosts and progression of disease (Kohler and Dobrindt, 2011). Iron limitation of Fe^3++^ was apparent from exposure to spinach root exudates, resulting in induction of systems for ferric iron and haem transport, via the enterobactin siderophore and Chu transport system respectively, while the ferrous iron transport system (*feo*) was repressed. The same limitation was not obvious in the other extracts, although there was some evidence for enterobactin production and transport on exposure to lettuce CWPS. Differences in access to extracellular and intracellular iron were evident, from induction of iron storage systems in spinach leaf lysates, in particular the ferritin protein FtnA (Andrews et al., 2003). Induction of the IscR Fe–S cluster assembly and repair system in the presence of lettuce CWPS supports previous data for exposure to lettuce (Kyle et al., 2010) or leaves of living plants (Linden et al., 2016), whereas the system was either un-induced or repressed in spinach extracts.

The biochemical analysis of the extracts coupled with the whole transcriptome analysis support a scenario in which *E. coli* O157:H7 (Sakai) adapted toward vegetative growth in the spinach leaf lysates, but could not grow and underwent multiple stress responses in the spinach root exudates. A likely possibility for the lack of available carbohydrates in the spinach root exudate preparations was depletion by native, ‘contaminating’ bacteria (Kuijken et al., 2015). Despite multiple attempts, it was not possible to remove these bacteria, and in our hands at least, they continue to be associated with spinach (see Materials and Methods).

Motility and adherence are important phenotypes that mark the initial stages of interaction with host tissue (Holden and Gally, 2004; Rossez et al., 2015). Induction of flagella genes in response to lettuce CWPS suggests that there is a signal for induction in the plant cell walls. This is consistent with the observation of flagella-mediated binding of *E. coli* to ionic lipids in the plasma membrane underlying the cell wall (Rossez et al., 2014b). Curli fibers are associated with biofilm formation and a switch from a motile to a sessile lifestyle is normally indicated by down-regulation of flagella genes and upregulation of curli genes (Pesavento et al., 2008). Such cross-regulation was evident from gene expression on exposure to root exudates consistent with a switch to sessility, i.e., repression of FlhDC, induction of curlin and Bdm. On the other hand, *E. coli* O157:H7 (Sakai) cells in CWPS were either motile or in transition, with some expression of *flhD* and *csgE*. Production of curlin fibers has been linked to colonization of fresh produce (Patel et al., 2011; Macarisin et al., 2012) and starvation conditions have been shown to induce a shift to a curli+ phenotype in plant-associated *E. coli* O157:H7 isolates (Carter et al., 2011). Bdm, a biofilm modulatory protein, is also linked to control of flagella genes, although the mechanism is as yet unclear (Kim et al., 2015).

The growth potential of *E. coli* O157:H7 (Sakai) on living plants differed to that seen in the plant extracts, which may reflect a contribution of host-derived factors. Whereas adaptation to the plant environment was only assessed during the initial stages of the plant–microbe interaction and *E. coli* O157:H7 (Sakai) was capable of growth in the extracts (with sufficient C-source), colonization potential reflects the capacity for the bacteria to become established on the plant. Growth in extracts to similar levels was demonstrated for *E. coli* O157:H7 (EDL933) in leaf lysates (Kyle et al., 2010). However, multiple factors are likely to impact the interaction including the complexity of the environment (i.e., how the plants are grown); accessibility of plant-derived metabolites; and the presence of an active host defense response, as had been reported for a number of plant-associated bacteria (Rosenblueth and Martinez-Romero, 2006; Holden et al., 2009; Hunter et al., 2010; Bulgarelli et al., 2012; Gutiérrez-Rodríguez et al., 2012; Hol et al., 2013; Turner et al., 2013).

Higher numbers of *E. coli* O157:H7 (Sakai) were recovered from the roots compared to the phyllosphere since the rhizosphere is a more hospitable environment protected from desiccation and UV irradiation that occur above ground, and has been reported to support substantially higher levels of other human pathogens (Brandl et al., 2004; Kroupitski et al., 2011). In general, higher levels of persistence were observed on the abaxial surfaces of leaves, which is also likely due to differences in UV irradiation and desiccation (Brandl, 2006). *E. coli* O157:H7 (Sakai) has previously been shown to have a propensity to bind to guard cells (Rossez et al., 2014b) and it is possible that differences in stomata density and distribution (Willmer and Fricker, 1996) may also influence the differences in the number of bacteria recovered. The reduction in numbers of *E. coli* O157:H7 (Sakai) on either surface of *L. serriola* may be due to the levels of polyphenols (Chadwick et al., 2016), which are associated with antimicrobial activity (Bach et al., 2011). *E. coli* O157:H7 (Sakai) was recovered in the highest numbers from the roots of plants grown under hydroponics conditions, which have a substantially reduced or absent native microbiota, suggesting that microbial competition is also an important factor in successful colonization.

Together, the data illustrate a complex interaction between RTE crop plants and *E. coli* O157:H7 that is dependent on ‘system’-specific differences. Metabolism was found to be an important bacterial driver of the initial stages of the interaction. It is possible that some of the uncharacterized genes (annotated as hypothetical) that were strongly regulated on exposure to plant extracts play an important role in bacterial colonization of plants. Furthermore, the differences in bacterial growth in extracts compared to longer-term persistence on live plants indicate that plant and/or environmental factors also influence the interaction. The fact that the plant species and tissue type have a strong influence on the initial bacterial response as well as the potential for colonization provides information that can contribute to predictive modeling or risk-based analysis of the potential for microbial contamination of horticultural crops.

## Materials and Methods

### Bacterial Strains and Growth Conditions

*Escherichia coli* O157:H7 strain Sakai (RIMD 0509952; Dahan et al., 2004) *stx^-^* Kan^R^ was used for all experiments. The bacteria were grown overnight in Luria-Bertani broth (LB broth) at 37°C, 200 rpm supplemented with 25 μg/ml kanamycin. For growth curve experiments and colonization assays, the bacterial overnight culture was sub-cultured in a 1:100 dilution into MOPS medium (10x MOPS solution: 0.4 M MOPS, pH 7.4; 0.04 M tricine; 0.1 mM FeSO_4_; 95 mM NH_4_Cl; 2.76 mM K_2_SO_4_; 5 mM CaCl_2_; 5.28 mM MgCl_2_; 0.5 M NaCl; 10 ml micronutrients [3 μM (NH_4_)_6_MO_7_O_24_H_2_O; 0.4 mM H_3_BO_3_; 0.03 mM CoCl_2_; 0.01 mM CuSO_4_; 0.08 mM MnCl_2_; 0.01 mM ZnSO_4_]; 0.2% glycerol; 132 mM K_2_HPO_4_; 0.02 M thiamine HCl; 50x essential amino acids and 100x non-essential amino acids (Sigma-Aldrich, St. Louis, MO, USA) at 18°C, 200 rpm until stationary phase. For all microarray experiments, the bacteria were subsequently sub-cultured into M9 minimal medium [20 ml 5x M9 salts (5x M9 salts): 64 g of Na_2_HPO_4_.7H_2_O; 15 g of KH_2_PO_4_; 2.5 g of NaCl; 5 g of NH_4_Cl; dissolved in 1 L sterile distilled water (SDW); 2 mM MgSO_4_; 0.1 mM CaCl_2_; 0.2% glycerol; pH 7.0] at 18°C and 200 rpm, unless otherwise stated.

### Growth Curves

*Escherichia coli* O157:H7 (Sakai) cultures were grown to saturation (∼18 h) at 18°C in M9 medium (as above) and diluted to an optical density of 0.02 (OD_600_) in M9 medium supplemented with 40% plant extracts, at 18°C and 200 rpm. Extracts were normalized for protein content to a concentration of 1 μg/ml total protein using a Bradford assay using the Micro BCA^TM^ Protein Assay kit (Thermo Scientific, Waltham, MA, USA) according to the manufacturer’s instructions. 1 ml of culture was taken at each time point and measured in a spectrophotometer at OD_600_. Samples were set up in triplicate and triplicate readings were taken for each. BSA standards were used to generate a standard curve for comparison.

### Colonization Assays

#### Leaves

Plants were grown in compost (containing peat, sand, limestone, perlite, calcite, Sincrostart, and Multicote 4) at 75% humidity, light intensity of 150 μmol m^2^ s^-1^ (16 h photoperiod: day temperature of 26°C, night temperature of 22°C) for three to 4 weeks. The bacterial culture was washed and re-suspended in phosphate buffered saline (PBS) at an OD_600_ of 1.0 (equivalent to ∼1x 10^8^ CFU/ml). A soft marker pen with indelible ink was used to mark 1 mm spots on to the adaxial and abaxial sides of the leaves (separate leaves were used for each). Two leaves were taken per plant, with three technical replicates of each taken in total. 2 μl of the bacterial culture was applied to the spot and left to dry for 1 h. Two μl of sterile PBS was pipetted onto the spots for un-inoculated, control plants. At each time point, leaves were excised, weighed and macerated in 1 ml PBS. The samples were diluted to 10^-3^ and plated onto Sorbitol-MacConkey (SMAC) agar containing 25 μg/ml kanamycin, incubated at 37°C (∼20 h) and the colonies counted the following day. The microbiological count data was calculated based on the fresh weight of each leaf and standardized as CFU per gram fresh tissue. Three biological repeats of the experiment were carried out. The data was transformed (log_10_) and analyzed by ANOVA using the Tukey multiple correction test (GraphPad Prism, version 5.0).

#### Roots of Compost-Grown Plants

Plants were grown as for the leaf colonization assay. The bacterial culture was diluted to an OD_600_ of 0.02 (∼1.6 × 10^7^ CFU ml^-1^) in 1 L of SDW. The plants were not watered for the preceding 24 h and were inoculated by partly immersing their pots in the bacterial suspension for a period of 1 h. Uninoculated negative control plant pots were immersed in 1 L SDW. At each time point, the roots were detached, washed gently in 20 ml PBS to remove the compost and weighed. The roots were then macerated and processed as for the leaves. Three biological repeats of the experiment were carried out. Data was analyzed as for the leaf colonization assay.

#### Roots of Hydroponics-Grown Plants

Seeds were surface sterilized with 2% (w/v) calcium hypochlorite (CaCl_2_O_2_) and germinated on distilled water agar. Seedlings were grown under aseptic conditions in 300 ml hydroponic pots containing 10 g of sterilized perlite with 10 ml of 0.5x Murashige and Skoog (MS) media with no added sucrose. The bacterial culture was washed and re-suspended in fresh 0.5x MS at an OD_600_ of 0.02. The 10 ml of 0.5x MS was removed from the hydroponic pots and replaced with 10 ml of bacterial suspension. The plants were left for 1 h before the first time-point. Uninoculated negative control hydroponic pots had only 0.5x MS solution added. At each time point, the roots were excised and processed as for the leaf colonization assay. A bacteria-only control in 0.5x MS, with no plant, did not show any growth of bacteria. Data was analyzed as for the leaf colonization assay.

#### Plant Extract Preparation

Spinach cv. Amazon (*Spinacia oleracea*), lettuce cv. Salinas (*L. sativa*), prickly lettuce (*Lactuca serriola*), and vining pea (*Pisum sativum)* were used in this study. Plants were grown in compost for 3–4 weeks for leaf lysate extract preparation. The leaves were removed, snap frozen in liquid nitrogen and ground to a fine powder. 10 g of the leaf powder was re-suspended in 40 ml SDW and centrifuged for 15 min at 5,000 × *g*. The supernatant was heated at 50°C for 30 min and clarified by centrifugation at 5,000 × *g* for 20 min and the final supernatant passed through a 0.22 μm sterile filter.

For root exudate extracts, seeds were first surface sterilized using 2% (w/v) calcium hypochlorite (CaCl_2_O_2_) for 15 min and germinated on distilled water agar. Seedlings were transferred to hydroponic pots containing 10 g rockwool and sterile 0.5x MS (no sucrose). After 3 weeks growth, the exudates were removed from 24 plants by three successive aqueous extractions with 50 ml SDW and clarification through a 0.22 μM filter. Spinach-associated bacteria were isolated on LB agar at room temperature, crude whole cell lysates prepared and subject to PCR for the 16 rRNA genes. The variable 2, 3, and 6 regions were sequenced and the isolates tentatively identified from BLAST analysis of the DNA sequence.

To prepare leaf CWPS extracts, plants were grown in vermiculite (William Sinclair Holdings, Lincoln, UK) containing Osmocote Start 6-weeks short-term base fertilizer for 3–4 weeks. The leaves were excised and macerated to a fine powder in liquid nitrogen. 10 g of the leaf powder was re-suspended in 40 ml SDW and the debris pelleted by centrifugation for 15 min at 5,000 × *g*. The plant powder was processed to obtain the alcohol insoluble residue (Popper, 2011). Briefly, 70% ethanol was added to the plant powder in a 5:1 ratio and mixed for 10 min at 80 rpm. The samples were pelleted by centrifugation at 5,000 × *g* for 10 min and the supernatant discarded. Ethanol extraction was repeated five times. 100% acetone was then added to the powder and mixed at 80 rpm for 10 min. The acetone wash step was repeated twice. Following this, the supernatant was discarded and the polysaccharide powder was left to air dry for 48 h. A no-plant vermiculite-only negative control was prepared using the same method to account for any residual carry-over from the vermiculite and serve as a base-line to assess gene expression.

### Plant Extract Inoculation for Whole Transcriptome Analysis

#### Temperature

*Escherichia coli* O157:H7 (Sakai) was grown in M9 minimal media at either 37 or 18°C until early stationary phase (OD_600_ of ∼1). Each culture was washed in M9 once and sub-inoculated to an OD_600_ of 0.5 in fresh M9 media with 0.2% glycerol, which had been preheated to 37 or 18°C. The cultures were incubated for 1 h at 37 or at 18°C, with aeration (200 rpm). After 1 h, the cultures were harvested for RNA isolation by mixed with RNA Protect (Qiagen).

#### Leaf Lysates/Root Exudates

*Escherichia coli* O157:H7 (Sakai) diluted to an OD_600_ of 0.5 into fresh M9 medium supplemented with 40% (v/v) spinach leaf lysate or root exudate extract (normalized to 1 μg/ml total protein content). Cultures were incubated at 18°C with aeration (200 rpm) for 1 h, harvested and mixed 1:1 with RNA Protect (Qiagen). *E. coli* O157:H7 (Sakai) grown at 18°C in M9 media with 0.2% glycerol without any plant extracts was used as the *in vitro* control and served as a base-line for gene expression.

#### Leaf Cell Wall Polysaccharides

*Escherichia coli* O157:H7 (Sakai) was diluted at an OD_600_ of 0.5 into fresh M9 medium with one of three supplements: 1% (w/v) spinach *S. oleracea* leaf CWPSs; or 1% (w/v) lettuce (*L. sativa*) leaf CWPSs (normalized to 1 μg/ml total protein content); or 1% (w/v) vermiculite no-plant control extract, and incubated at 18°C, 200 rpm for 1 h and processed as for the leaf lysates experiment.

#### RNA Extraction

Total RNA was extracted from samples stored in RNA Protect using the RNeasy Plant Mini kit RNA extraction protocol (Qiagen). The concentration of total RNA was estimated using a NanoDrop (Wilmington, MA, USA) spectrophotometer and visualized for quality using a Bioanalyzer 2100 (Agilent Technologies, Santa Clara, CA, USA). Genomic DNA carryover was removed using the TURBO DNA-free kit (Ambion, Life Tech) and verified as DNA-free from a negative PCR reaction using *gyrB* primers, compared to a positive control.

#### Microarray Processing and Analysis

The complete microarray experimental plan and datasets are available at ArrayExpress (^1^accessions #E-MTAB-3249 and E-MTAB-4120). Microarray processing was essentially performed as described for other prokaryotic species (Venkatesh et al., 2006). Briefly, cDNA synthesis was performed using Superscript reverse transcriptase (Invitrogen) and labeled with either Cy3 or Cy5 dye according to the microarray plan. The Agilent microarray used (Agilent #G4813A-020097; accession # A-GEOD-8701) contains 15,208 probes representing transcripts from a total of four genomes: *E. coli* MG1655; *E. coli* CFT073; *E. coli* O157:H7 EDL933; and *E. coli* O157:H7 Sakai. A single color approach was used for the temperature, leaf lysate and root exudate conditions. Four replicate samples of each of the four conditions [*E. coli* (Sakai) in: (i) M9 media at 18°C; (ii) M9 media at 37°C; (iii) M9 media plus spinach leaf lysate at 18°C, and; (iv) M9 media plus spinach root exudate at 18°C] were run. For the polysaccharide conditions, a two-color approach was used. Eight replicate samples of the control condition [*E. coli* (Sakai)] in M9 media with vermiculite extract at 18°C were labeled as detailed, along with four replicates of the two test conditions [*E. coli* (Sakai)] in M9 media with spinach/lettuce leaf CWPSs. Labeled cDNA was hybridized to microarrays as recommended by the manufacturer. Microarrays were scanned using a G2505B scanner (Agilent) and data extracted from images using Feature Extraction software (Agilent v. 10.7.3.1) with default parameters. Data were subsequently imported into GeneSpring GX 7.3 (Agilent, USA). Quality control was applied to remove those probes with no consistent signal in any of the conditions tested, whereby data was filtered on flags being present or marginal in two out of the three replicate samples. Principal component analysis was performed to identify any outliers. For all microarray experiments, statistical analysis of the datasets was carried out by performing a Volcano plot on each condition with a twofold minimum cut off for fold change and a Student’s *t*-test with multiple testing correction (Benjamini and Hochberg; *p* ≤ 0.005 for temperature, spinach leaf lysates and spinach root exudate conditions; *p* ≤ 0.01 for lettuce polysaccharide; *p* ≤ 0.05 for spinach polysaccharide). Filtering was carried out in Microsoft Excel on raw values from the array pixel density (>50), and where multiple probes represented the same gene: as a consequence of the array design genes are represented with 1–4 probes for the four strains MG1655 (‘b’ accession number prefix), CFT073 (‘c’), TUV93-0 (‘Z’), and Sakai (‘ECs’). Data for duplicate probes were removed to provide data preferentially for ECs or Z, followed by b accession numbers. Metabolic pathway analysis was performed using EcoCyc^2^ (Keseler et al., 2013). GO enrichment analysis was performed from the Gene Ontology Consortorium website (The Gene Ontology Consortium, 2015), using the PANTHER classification system (Mi et al., 2016) for Biological Processes (GO-Slim and GO-Complete), and only classes with significant enrichment (*p* < 0.05) were analyzed. Blastn analysis was carried out at the NCBI database (Altschul et al., 1990).

#### HPLC Analysis

Leaf lysate and root exudate extracts were prepared for HPLC by ethanol extraction. 10 ml of samples were freeze dried and re-suspended in 80% ethanol. The mixture was centrifuged at 5,000 × *g* for 30 min. The supernatant was collected, and freeze dried once more after ethanol evaporation before being re-suspended in 2 ml molecular biology grade water. Leaf CWPS samples were prepared by TFA hydrolysis. Briefly, 10 mg of polysaccharide samples was incubated with 2 M trifluoracetic acid and boiled at 100°C for 1 h. The TFA was removed by evaporation and the sample freeze dried before re-suspending in 1 ml of molecular biology grade water. Samples were run on a Dionex chromatography machine with the Chromeleon software using a PA100 column for glucose, fructose, sucrose, arabinose, and rhamnose.

#### Quantitative Reverse Transcriptase (qRT) PCR Analysis and Microarray Data Validation

All qRT-PCR reactions were set up with iTaq^TM^ Universal SYBR© Green Supermix (Bio-Rad) according to manufacturer’s instructions, with 300 nm of primer and run in a Step-One Plus machine (Applied Biosystems) using the ΔΔCt method with an additional melt-curve analysis. All primers were validated as having 95–100% efficiency prior to ΔΔCt analysis, similar to that of the reference gene. Reference genes were validated using the GeNorm kit and software (Primer Design, Southampton, UK), for which g*yrB* was used as it was stably expressed under all microarray conditions (M > 0.1). qRT-PCR data was analyzed by averaging three technical and three biological replicates and applying the formula 2^-ΔΔCt^, with the data normalized to the calibrator sample and to the validated reference gene. Microarray expression data was validated by examining the expression of 18 genes by qRT-PCR and measuring the correlation coefficient between both datasets for relevant subsets of these genes (i.e., significantly up or down-regulated). This was done for the microarrays samples and for an independent set of samples. The correlation coefficients (*R*^2^) were 0.9994; 0.9851; 0.9160; 0.9730; 0.9201 for the temperature; spinach leaf lysate; spinach root exudate; spinach CWPS; and lettuce CWPS treatments, respectively.

## Author Contributions

LC: acquisition, analysis, and interpretation of the data; drafting and revision of the m/s; PH and JM: design, acquisition, and analysis of microarray data; drafting the m/s; CW: provision of *L. serriola*; design of the colonization experiments; drafting the m/; SA and IT: design of microarray and colonization experiments; drafting and revising the m/s; data interpretation; RJ: conception and design of the work; drafting and revision of the m/s; NH: conception and design of the work; analysis and interpretation of the data; drafting and revision of m/s; all: final approval; agreement for accountability.

## Conflict of Interest Statement

The authors declare that the research was conducted in the absence of any commercial or financial relationships that could be construed as a potential conflict of interest.

## Footnotes

1 https://www.ebi.ac.uk/arrayexpress/

2 http://ecocyc.org

## Supplementary Material

FIGURE S1 **GO term enrichment for response to temperature.** GO terms for *E. coli* O157:H7 (Sakai) genes that were significantly differentially expressed following growth in M9 medium at 18°C relative to 37°C. Data was obtained from the Gene Ontology Consortium website. Significantly enriched Biological Processes are shown for induced (blue) and repressed genes (red), using GO-Slim and selected GO-complete categories (indicated by ‘^∗^’; full list in Supplementary Table S2). The numbers of individual genes are adjacent to each bar on the charts.

FIGURE S2 **Validation of *E. coli* O157:H7 (Sakai) gene expression from the microarray results by qRT-PCR.** Expression of *malE* **(A)** and *dctA* **(B)** in response to CWPSs was compared for the microarray probes and from two separate sets of RNA extractions for the qPCR analysis (qPCR.1, qPCR.2). The no-plant control is designated ‘Control’ and either spinach or lettuce CWPSs by ‘PS.’ Numbers represent the average of nine technical replicates for each of the qPCR datasets; data was analyzed using the ΔΔCT method by comparing to a validated housekeeping gene (GeNorm), using primers of equal (95–100%) efficiency.
