# Corrigendum: Whole-Transcriptome Analysis of Verocytotoxigenic *Escherichia coli* O157:H7 (Sakai) Suggests Plant-Species-Specific Metabolic Responses on Exposure to Spinach and Lettuce Extracts

**DOI:** 10.3389/fmicb.2016.01506

**Published:** 2016-09-21

**Authors:** Louise Crozier, Pete E. Hedley, Jenny Morris, Carol Wagstaff, Simon C. Andrews, Ian Toth, Robert W. Jackson, Nicola J. Holden

**Affiliations:** ^1^Cell and Molecular Sciences, The James Hutton InstituteDundee, UK; ^2^School of Chemistry, Food and Pharmacy, The University of ReadingReading, UK; ^3^School of Biological Sciences, The University of ReadingReading, UK

**Keywords:** DNA microarray, stress response, *E. coli* O157:H7, vegetables, leaves, roots, adaptation, biological

In the Introduction to the article, one of the references, Kyle et al. (2010) was included in error in the statement “However, in many reports on plant-colonization transcriptomics the bacteria were initially cultured at body temperature (37°C) and were subsequently exposed to plant (or plant extracts) at environmental temperature (~18°C); such experimental regimes result in a considerable temperature shift, in addition to the exposure to plant or plant extracts (Thilmony et al., 2006; Kyle et al., 2010; Hou et al., 2012, 2013; Jayaraman et al., 2014)”. Instead, the Kyle et al. study used a temperature of 28°C throughout the experiment, for initial culturing of the inoculum and for subsequent bacteria-plant interactions, so that the bacteria did not encounter a temperature shift. An extract from the Methods section is provided below. Erroneous inclusion of this reference in the statement has no impact on the scientific validity of the results presented.

The correct statement is: “However, in many reports on plant-colonization transcriptomics the bacteria were initially cultured at body temperature (37°C) and were subsequently exposed to plant (or plant extracts) at environmental temperature (~18°C); such experimental regimes result in a considerable temperature shift, in addition to the exposure to plant or plant extracts (Thilmony et al., 2006; Jayaraman et al., 2014; Hou et al., 2012, 2013)”

Extract of the Methods section from Kyle et al. (2010):

“For experiments measuring early gene expression in lettuce leaf lysate (by microarray and QRT-PCR) or on shredded lettuce (by QRT-PCR), overnight cultures were transferred to fresh M9-glucose and grown for several hours into the mid-log phase of growth at 28°C and 150 rpm and then washed twice with KP buffer before inoculation. The lysates were inoculated with EcO157 cells in the mid-log phase of growth in minimal medium in order to isolate the bacterial responses to romaine lettuce lysates from changes in gene expression solely caused by the transition out of stationary phase (6). Lysates were inoculated at 5 × 10^6^ CFU/ml for growth experiments and at 10^8^ CFU/ml for microarray analysis and QRT-PCR and then incubated at 28°C with shaking at 150 rpm. In order to evaluate gene expression in EcO157 in lettuce lysates, samples for RNA extraction and subsequent microarray or QRT-PCR analysis were taken at 15 or 30 min after exposure of mid-log-phase EcO157 cells to freshly prepared lysates. Short incubation periods in the lysates at 28°C were used in order to characterize the early response of the pathogen to fluids leaking out of leaf cells after injury occurred, at an ambient daytime temperature that would be present in the field during growth and harvesting, or during processing under conditions that would fail to maintain cool temperatures.”

## Author contributions

NH: wrote the correction statement. LC, PH, JM, CW, SA, IT, RJ, and NH: approved statement.

### Conflict of interest statement

The authors declare that the research was conducted in the absence of any commercial or financial relationships that could be construed as a potential conflict of interest.
