# Supplementary figures and images for: Whole-Transcriptome Analysis of Verocytotoxigenic Escherichia coli O157:H7 (Sakai) Suggests Plant-Species-Specific Metabolic Responses on Exposure to Spinach and Lettuce Extracts
Source: Front Microbiol. 2016 Jul 12;7:1088. doi: 10.3389/fmicb.2016.01088 (PMC4940412; doi:10.3389/fmicb.2016.01088)

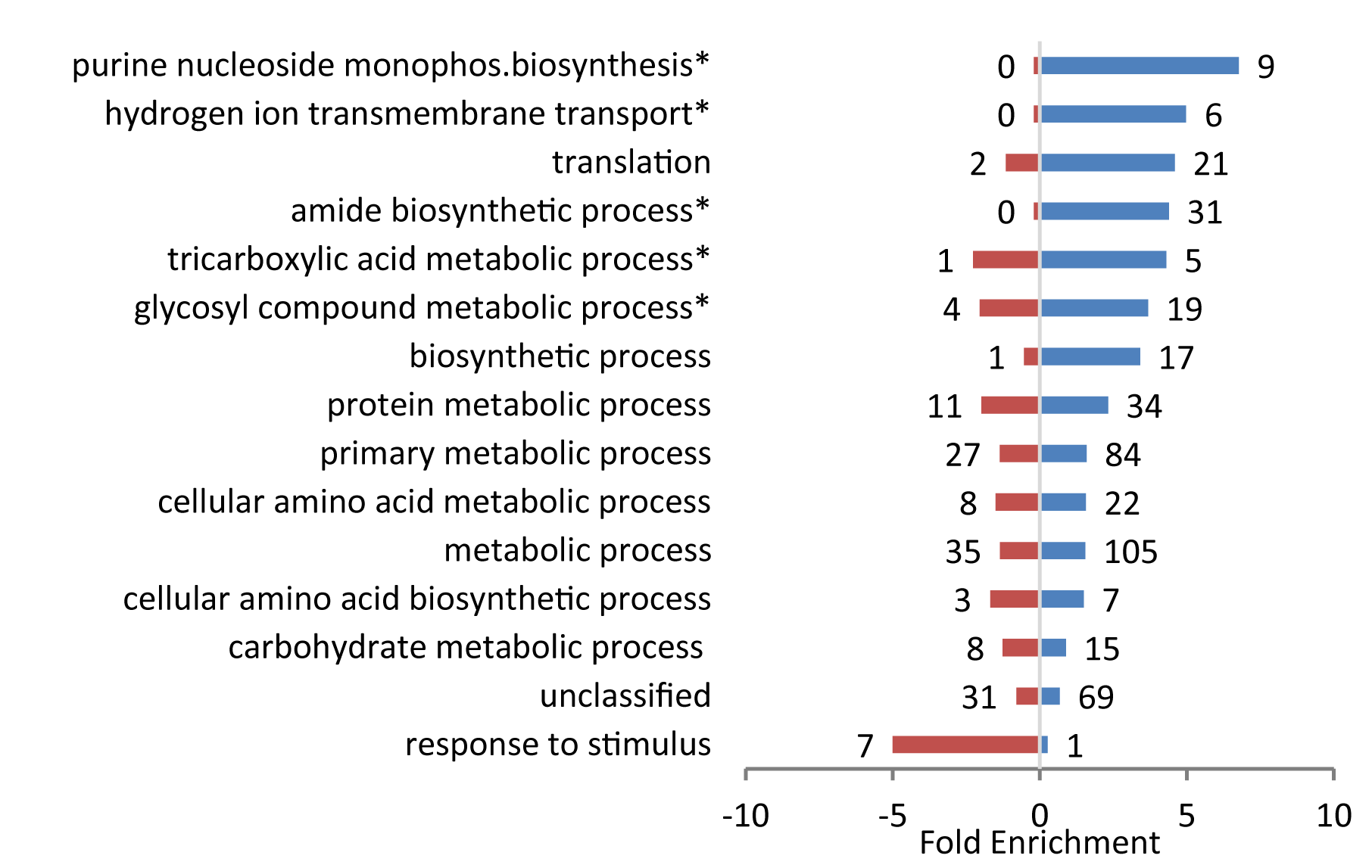

Supplement: FIGURE S1 — GO term enrichment for response to temperature. GO terms for E. coli O157:H7 (Sakai) genes that were significantly differentially expressed following growth in M9 medium at 18°C relative to 37°C. Data was obtained from the Gene Ontology Consortium website. Significantly enriched Biological Processes are shown for induced (blue) and repressed genes (red), using GO-Slim and selected GO-complete categories (indicated by ‘∗’; full list in Supplementary Table S2). The numbers of individual genes are adjacent to each bar on the charts. [file Image_1.TIF]

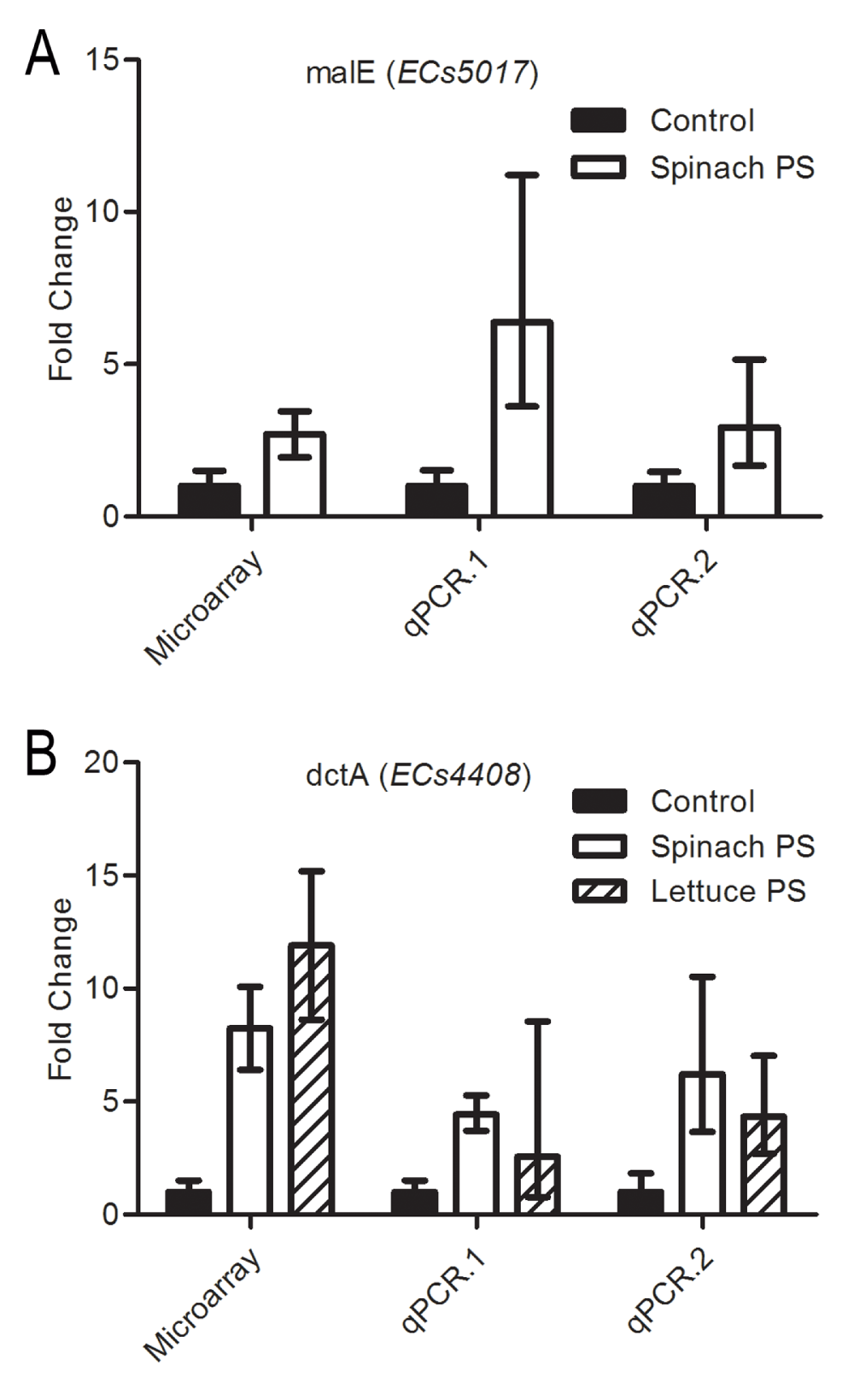

Supplement: FIGURE S2 — Validation of E. coli O157:H7 (Sakai) gene expression from the microarray results by qRT-PCR. Expression of malE (A) and dctA (B) in response to CWPSs was compared for the microarray probes and from two separate sets of RNA extractions for the qPCR analysis (qPCR.1, qPCR.2). The no-plant control is designated ‘Control’ and either spinach or lettuce CWPSs by ‘PS.’ Numbers represent the average of nine technical replicates for each of the qPCR datasets; data was analyzed using the ΔΔCT method by comparing to a validated housekeeping gene (GeNorm), using primers of equal (95–100%) efficiency. [file Image_2.TIF]
